# Implementing a novel strategy for interprofessional medication review using collegial mentoring and systematic clinical evaluation in nursing homes (COSMOS)

**DOI:** 10.1186/s12877-019-1139-6

**Published:** 2019-05-07

**Authors:** Christine Gulla, Elisabeth Flo, Reidun L. S. Kjome, Bettina S. Husebo

**Affiliations:** 1 Centre for Elderly and Nursing Home Medicine, Department of Global Public Health and Primary Care, University of Bergen, Bergen, Norway; 2 Department of Clinical Psychology, University of Bergen, Bergen, Norway; 3 Department of Global Public Health and Primary Care/Centre for pharmacy, University of Bergen, Bergen, Norway

**Keywords:** Medication review, Nursing homes, Neuropsychiatric symptoms, Implementation, Symptom assessment, Needs assessment, Behavioral and psychological symptoms of dementia

## Abstract

**Background:**

Multimorbid patients in nursing homes are prescribed long lists of medication, often without sufficient clinical evaluations beforehand. This results in poor clinical effects of the prescribed medication and significant side-effects, especially in patients with impaired cognition. The aim of this paper is to describe the process, content and implementation of a clinical medication review encompassing clinical testing and collegial support to prescribers.

**Methods:**

The implementation process of a novel approach to medication review in nursing homes was logged thoroughly by structured staff feedback. Staff experienced promotors and barriers to implementation also were collected. The study was part of a cluster randomized controlled trial, in which 36 long-term care units received the COSMOS intervention. Nurses and physicians randomized to the intervention group participated in educational programs, training in clinical evaluation of the patients, and interprofessional medication review with collegial mentoring.

**Results:**

The intervention group contained 297 patients from 36 nursing home units. There were 105 staff attendees for the education program. The units were served by 21 different physicians. Clinical medication reviews were performed in all units and all patients were assessed prior to the medication reviews. Of the 240 patients with a logged intervention process, 220 (92%) underwent a medication review. The intervention generated enthusiasm and improved communication among nursing staff and between nursing staff and physicians. The interprofessional discussions helped to facilitate difficult decisions pertaining to treatment levels. Reported barriers were lack of time, low engagement of all nursing staff and physicians, and ethical dilemmas.

**Conclusions:**

Clinical medication reviews were implemented for almost all patients, and every patient was systematically assessed prior to the medication review. The physicians perceived collegial mentoring as an asset, learning from each other facilitated decision making in terms of difficult aspects of prescribing. Knowledge about barriers and promotors can improve implementation of similar interventions in other nursing homes.

**Trial registration:**

Clinicaltrials.gov (NCT02238652). Registered July 7th 2014.

## Background

Nursing home patients are old and fragile, and over 80% have dementia [1]. Multimorbidity is common in old age, and cardiovascular diseases, stroke, cancer, and psychiatric disorders often co-occur [2, 3]. A consequence of multimorbidity is polypharmacy, affecting most nursing home patients, who on average use eight different drugs every day, and two on demand [4]. The altered pharmacokinetics and pharmacodynamics of the aged body increase susceptibility to adverse events from drugs [5]. A study by Soraas et al. (2014) found that the use of eight or more drugs significantly increased the risk of drug-drug interactions [6], which in turn leads to falls, cognitive decline, medication-related problems, and even increased mortality [7–10].

In nursing homes, physicians frequently prescribe drugs without a proper clinical evaluation of the patient [11]. Dementia reduces the patient’s ability to report effects and side effects of the treatment and depend on a proxy-rater who has known the patient over time to conduct a medical evaluation. It has been suggested that a systematic review of medications might be a procedure that will help ensure safe and appropriate medical treatment. Randomized controlled trials in nursing homes have been used to test medication reviews including: expert advice, the use of explicit prescribing lists, and multidisciplinary teams with the general practitioner (GP) involved [12–14]. They all demonstrated a reduction in the number of drugs without a detrimental effect on the patient’s health. Other approaches have been tested to improve drug prescribing by involving: a pharmacist, electronic prescribing aides, and the use of explicit prescribing criteria [15–17]. The interventions often comprise of multiple components, require new knowledge and involve different professions; it has been demonstrated that these elements impede implementation [18].

To ensure that complex interventions are successful and sustainable over time, different approaches have to be combined, and the implementation process must be planned and described in detail. Yet, multicomponent intervention studies have fallen short in reporting their implementation strategies and evaluation there-of [16]. Consequently, lack of efficacy may be caused by poor implementation, the ineffectiveness of the method or a combination of the two. We believe that we can improve prescribing and the medication review procedure in nursing homes by incorporating clinical assessment using tools validated for people with dementia, and by testing to ascertain whether the intervention was carried out successfully.

In this paper, we describe a novel implementation strategy for an interprofessional medication review. It is based on systematic clinical evaluation of the patient and collegial mentoring of the nursing home team. We describe the process of implementation and report the findings for the following research questions:

1. How did nursing home staff receive the intervention?
2. To what degree was the medication review implemented successfully?
3. What are the barriers and facilitators for implementing interprofessional medication reviews in nursing homes?

## Method

### Data source

The medication review was a part of the COSMOS study, a 9-month multicenter, cluster randomized controlled trial [19]. The trial used a multicomponent intervention, consisting of five elements represented by the acronym COSMOS: *CO*mmunication, *S*ystematic pain assessment and treatment, *M*edication review, *O*rganization of activities, and *S*afety. We have previously described the study protocol in detail [19], and the main outcomes [20]; the trial is registered at clinicaltrials.gov (NCT02238652).

For this study, we include data from the 36 Norwegian nursing home units that received the COSMOS intervention, covering 297 patients. The recruited nursing homes were located in small and large municipalities, in different geographical locations, and from more or less prosperous municipalities. We included long-term care units and specialized dementia wards with unit capacity varying from eight to 28 patients per unit. All patients living in or moving into the units during the first two months of the trial were eligible for inclusion if they had a minimum stay of two weeks before the first assessment. We excluded patients under 65 years of age, those with schizophrenia, and patients deemed by the nursing home physician to have less than six months to live. In general, medical treatment in Norwegian nursing homes is provided by physicians (mostly GPs) working one to five days a week in the nursing home. The proposed standard of care by the Norwegian Medical Association for Norwegian long-term care patients are 90 patients per full time physician [21].

### Implementation of the medication review

**Fig. 1:**
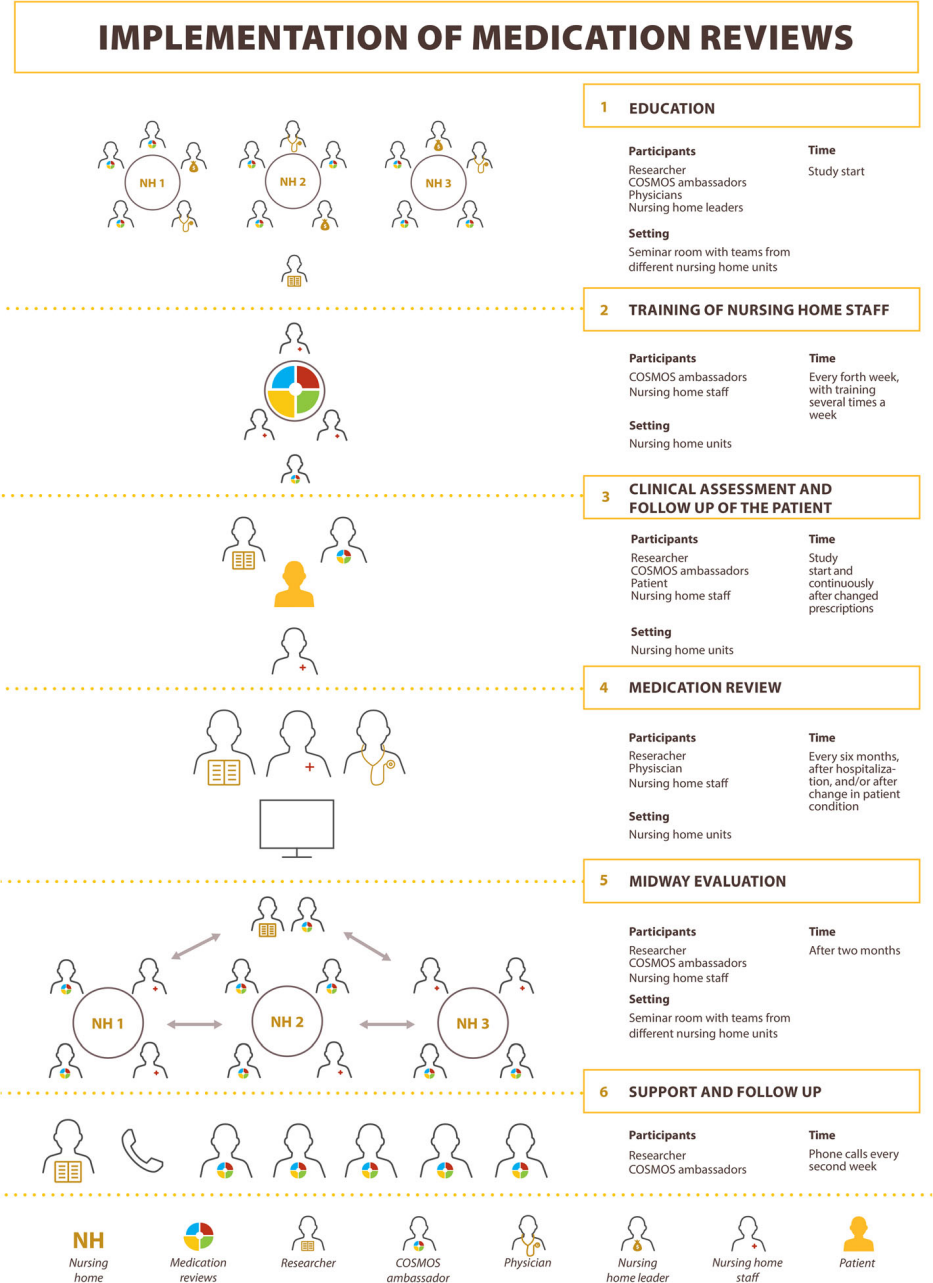
The medication review process: 1: Researchers educated nursing home staff (“COSMOS ambassadors”), physicians, and nursing home leaders in safe use of drugs and the medication review intervention. 2: The COSMOS ambassadors used the lessons from the education to train the other staff in their units. 3: The researchers trained the nursing home staff in assessing the patients’ pain, neuropsychiatric symptoms, cognition, daily function, and quality of life. 4: Multidisciplinary medication reviews with the researchers, nurses, and nursing home physician were performed in each unit. The researcher gave collegial mentoring for the other participants. The results from the clinical assessments were used in evaluation of the prescriptions for each patient. 5: After two months, the nursing home staff was gathered for a midway evaluation to discuss promotors and barriers towards the implementation among themselves and with the researchers. 6: During the whole study period, the researchers regularly called the COSMOS ambassadors to follow up on the implementation. The researchers gave advice on how to overcome barriers, and collected practical tips from the COSMOS ambassadors that could be spread to other units

The implementation consisted of six steps demonstrated in Fig. 1.

Step 1: Initially, nursing home managers, registered nurses, licensed practical nurses, unit managers, physicians, and pharmacists from the intervention nursing homes were invited to participate in a standardized education program (Fig. 1.1). The program started with a two-day seminar held by the researchers (BSH and EF). One prerequisite was that at least two nurses (named “COSMOS ambassadors”) from each nursing home unit, who had regular patient contact, had to participate in the program. The education consisted of four hours lecturing, role-playing, and problem-solving discussions on a) Pharmacodynamics, pharmacokinetics, multimorbidity, and clinical challenges; b) Importance of systematic clinical assessment, documentation and follow-up; c) Unnecessary drug use, and drugs with relevant adverse or anticholinergic effects; d) Drug-drug interactions; e) Training the unit’s staff and practical information about the medication review. The content of the education program was based on the Norwegian guidelines for medication review [22], the Norwegian Patient Safety Campaign [23], and literature reviews on medication reviews in nursing homes [16, 17, 24–27]. The drug recommendations were based on the Screening Tool of Older Persons Potentially Inappropriate Prescriptions/Screening Tool to alert Doctors to Right Treatment (START/STOPP) 2 criteria [28], and the Norwegian Medicines Agency’s checklist for medication reviews [29]. In addition, we adapted the anticholinergic drug list developed by Duran et al., to drugs available on the Norwegian market [30].

Step 2: After the initial 2-day seminar, the COSMOS ambassadors were responsible for training the rest of the unit’s staff in short session of about 10–20 min each (Fig. 1.2). We encouraged them to adapt the procedure to their local routines but made suggestions as to how they could organize the training. For instance, we advised the ambassadors to offer training during lunch and/or report several times a week to enable all nursing staff members to participate. This train-the-trainer approach was employed to ensure that the medication review implementation was sustainable, through involving the entire nursing home unit [31].

Step 3: Researchers trained the nursing home staff in assessing the patient’s pain, neuropsychiatric symptoms, cognition, daily function, and quality of life. At the start of the study, each patient was assessed using a range of instruments developed and validated for use in elderly patients and persons with dementia (Fig. 1.3, Table 1). To ensure the quality of the assessments, at least two registered nurses or licensed practical nurses underwent four hours of individual training in the use and interpretation of the instruments. The session was conducted in the patients’ units with practical bedside mentoring; for instance, the use of MOBID-2 was demonstrated during the morning care of at least two patients.

**Table 1:** Instruments used in assessment of patient prior to medication review

Short name	Range	Interpretation
Cognition		
MMSE	0–30	The patient is asked 30 questions, scored correct (1) or wrong (0). Lower scores indicates lower cognitive function [47]. Normal cognition was defined as: 26–30, mild dementia: 21–25, moderate dementia 11–20, severe dementia < 11 [48].
FAST^a^	1–7	Staging cognitive function, normal to severe dementia [49]. Normal cognition was scored as 1–2, 3–4 mild dementia, 5: moderate dementia, and 6–7 severe dementia.
Neuropsychiatric symptoms		
NPI-NH^a^	0–144	12 individual items: delusions, hallucination, agitation, depression, anxiety, euphoria, apathy, disinhibition, irritability, aberrant motor behaviour, night-time behaviour, and eating disturbances. Each item is scored by frequency (“absent” to “daily”; 0–4), and intensity for the patient (“mild” to “severe”; 1–3) these scores are multiplied to a sum score of 0–12 for each item and summed to give a total score [33, 50].
CMAI^a^	29–203	29 items with agitated behaviors (scored by frequency: “never” to “multiple times an hour”; 1–7) [51]. Agitation was defined as ≥39.
CSDD^a^	0–38	19 items on depressive behaviour (“absent” to “severe”; 0–2) [32]. Depression was defined as ≥8.
Pain		
MOBID-2^a^	0–10	Pain intensity during five standardized, guided movements, and five domains related to internal organs, head and skin during the last week, each item is scored 0 to 10 (“no pain” to “worst imaginable pain”). A total pain score is based on the worst pain experienced. A score of ≥3 signifies a need for pain treatment [52, 53]
Activities of daily living		
PSMS^a^	0–30	6 items of toileting, feeding, dressing, grooming, physical ambulation, and showering are scored whether the patient are able to do the activity or unable (0–5), higher scores indicate more dependency [54].
Quality of life		
QUALID^a^	11–55	11 items on patient behaviour rated on severeness; 1 to 5. Lower score indicates higher quality of life [55].

^a^Proxy-rated instrument. *CMAI* Cohen-Mansfield Agitation Inventory, *CSDD* Cornell Scale for Depression in Dementia, *FAST* Functional Assessment Staging, *MMSE* Mini Mental State Examination, *MOBID 2* Mobilization-Observation-Behaviour-Intensity-Dementia 2 Pain Scale, *NPI-NH* Neuropsychiatric Inventory- nursing home version, *QUALID* quality of life in late-stage dementia

Step 4: Medication reviews were performed by the nursing home physician together with the nurses and the researchers (BSH: anaesthesiologist, nursing home and palliative care physician; CG: physician), who provided collegial mentoring. The reviews were based on the results from the clinical assessments. We used these results to evaluate the necessity of the prescriptions for each patient (Fig. 1.4). The COSMOS guidelines instructed that all patients should have a medication review at least biannually, upon discharge from hospital and when their medical condition changed. Each patient’s general condition, prescriptions, and test results were discussed in detail. The drugs were assessed, individually and overall, in terms of all the patient’s conditions and life expectancy. For instance, if a patient scored 4 on the Cornell Scale for Depression in Dementia (CSDD) [32], and the Neuropsychiatric Inventory- nursing home version (NPI-NH) [33] item on depression was 4, both indicating no depression, and the patient was prescribed an antidepressant, we discussed whether the medication could be paused, or if there were reasons for continued use. The responsible physician executed the changes he or she saw fit based on the discussions. All changes in drugs were documented in the electronic patient record. The nursing staff re-assessed the patients` conditions after changes in drug treatment, and the physicians re-instated the drug if deemed necessary. Table 2 shows each participant’s responsibilities regarding the medication review.

**Table 2:** Responsibilities for the participants concerning the medication reviews

Participant	Responsibilities
Physician	Order relevant blood tests before the medication review. Already existing blood test results could be used if they were not older than two weeks. Medical decision-making, including choice of drug and dose.
Nurse	Prior to the meeting use www.interaksjoner.no [56] to identify potential drug-drug interactions for each patient. Report the patient’s condition and complaints, and close observation and follow-up after changes in treatment. Information to patients, relatives, and nursing staff about changes.
Researchers	Plan the meeting and compile the results from the baseline assessment of the patients on a spreadsheet. Guide the interprofessional team through the medication review. Provide collegial mentoring and updated knowledge. Give general information on the physician’s prescribing practice, such as average number of regular and on demand prescriptions, in comparison to national numbers

Step 5: After two months, nursing home staff was gathered for a midway evaluation to discuss promotors and barriers towards the implementation with the researchers. This was done by structured questions to each unit. They were asked to state three things they perceived they had succeeded with in the study, two things they had performed to some degree but still had some issues with, and one thing they had not performed at all or found difficult. These experiences were discussed in the group, and gave the staff the possibility to share experiences and learn from each other across the units (Fig. 1.5).

Step 6: COSMOS ambassadors were supported regularly (twice a month) by telephone contact (Fig. 1.6). Researchers gave advice on how to overcome barriers, and shared practical tips from the COSMOS ambassadors that could be spread to other units. The researchers counseled the nursing home staff on how to discuss medication changes with patients (if possible) and their relatives, who were informed about the medication review in advance. We recommended that if a drug was to be stopped, the physician and nurse should use the term “pause”. This ensured that the patient had to be reevaluated after the cessation and patient and relatives were less likely to interpret the change as a denial of treatment.

### Data collection and analyses

Patients were recruited and included in the study from August 1st 2014 through March 15th 2015. Patients were followed for four months, and the last follow up data were collected June 19th 2015. Data on the patients’ medical information and information on drug prescriptions were extracted from the patient’s medical record. Ongoing drug prescriptions were coded according to the fourth level of the Anatomical Chemical Therapeutic (ATC) Index [34]. Psychotropic drugs included the groups for antidepressants, antipsychotics, anxiolytics, hypnotics, and anti-dementia drugs. A questionaire about unit size and staffing was distributed to all unit managers. All attending physicians filled in another questionnaire to provide data on their gender, spesialization, employment, and number of patients they served.

Data on implementation was gathered using a “patient log” in which the primary nurse could document the implementation progress of every patient. The following five questions were answered (yes/no/not relevant): 1. Has a medication review been conducted? 2. Are all drug indications recorded? 3. Are changes in patient’s health documented? 4. Have any drugs been re-instated after withdrawal? 5. Is the patient (if mentally capable) and/or relatives informed about changes in the medication? In addition, the nurse had the option of writing comments. The researchers collected the logs at the end of the month-four data collection. Perceived barriers and promoters to the implementation were collected from the structured questions at the midway evaluation, we also gathered the remarks from the patient logs, as well as through feedback given to the researchers during medication reviews. These data were gathered in a database.

### Analyses

Demographics and clinical characteristics for the baseline population were presented with means and standard deviation (SD) or frequencies and percentages, as appropriate. All analyses were performed in IBM SPSS version 23, (Armork, NY). “To gain insight into the diversity of barriers and promotors, we chose a qualitative approach. We used a simple thematic analysis as described by N Mays and C Pope [35] to systematically examine these issues, to be able to identify promotors and barriers. Two researchers (CG and RLSK) read the transcribed feedback independently, and systematically searched for recurring themes of interest. We thematically coded the “main messages”, at first using the participants language, then gradually refining the codes and developing clearer categories through an iterative process of re-reading and discussion [35].”

## Results

Twenty of the 36 intervention units (56%) reported having had a focus on medication reviews in the three years preceding the intervention. Six units had participated in the Norwegian Patient Safety Campaign on improving prescribing in nursing homes [23], five had been a part of a research project focusing on medication review, and eight had a local focus on medication reviews. No pharmacists attended the medication reviews or the education, mostly because the units did not have any pharmacists on staff (Table 3).

**Table 3:** Experience, education and workload in health professionals in the intervention group

Nursing home units in nursing home, N	36 units in 18 NH	
Staffing, number of patients per nursing staff^a^ (range)	Daytime	3.2 (1.6–4.0)
	Evening	4.7 (2.3–6.0)
	Nighttime	13.0 (4.0–30.3)
*COSMOS ambassadors with direct patient contact, N (N per cluster)*		73 (2.0)
Registered nurses, N (%)		44 (61%)
Licensed practical nurses, N (%)		9 (12%)
Unknown education		19 (27%)
*Physicians*		21
Age in years, mean (SD)		48 (12.9)
Female, N (%)		8 (38%)
Mean number of patients in the study, per physician, mean (range)		22 (8–28)
Minutes per patient per week, mean (range)		20 (8–42)

^a^Nursing staff: Registered nurses, licensed practical nurses, and uneducated staff

*N* Number, *NH* nursing homes, *SD* Standard deviation

Twenty-one physicians had the medical responsibility for the nursing home units (Table 3). Seven physicians (33%) were full-time nursing home physicians and 14 (67%) were GPs with visiting hours at the nursing home. Thirteen had a specialization, 12 of which were in family medicine, and one in internal medicine. The nursing staff in the units consisted of registered nurses, licensed practical nurses, and staff without training. In 30 (45%) units, most staff were hired in 75–100% of full-time equivalent positions; in 24 units (36%), most staff held 50–75% of full-time equivalent positions.

The patients had a mean age of 87 (SD = 7.7) years, 73% were female, and they had a mean of 4 (SD = 3.3) registered diagnoses each. During the four months of the study, 33 patients died and 14 moved to another institution. All participants used a mean of 7.6 (SD = 3.8) drugs each day, ranging from 0 to 19, and had on average 3.4 (SD = 2.3) on demand prescriptions, ranging from 0 to 17. The most frequent regular drug groups were: laxatives (*N* = 172, 58%), antithrombotic agents, (*N* = 155, 52%), acetaminophen (*N* = 136, 46%), antidepressants (*N* = 118, 40%), and high ceiling diuretics (*N* = 95, 32%). The most frequent drugs on demand were acetaminophen (*N* = 147, 50%), anxiolytics (*N* = 134, 45%), opioids (*N* = 106, 36%), hypnotics (*N* = 82, 28%), and laxatives (*N* = 79, 27%).

### Education of nursing home staff

The education program was attended by 105 nursing staff. All units in the intervention group participated and all units sent the required two ambassadors, the average number was three participants per unit. The attendees were mainly two registered nurses and a unit manager or a licensed practical nurse for each unit. Seven of the 21 physicians attended the education program. The non-attending physicians cited limited time and lack of relevance as reasons for not attending. The same number of people attended the midway evaluations as the education program. These meetings had no management level staff or physicians present, but more regular nursing staff participated.

The training materials were clearly visible in all units during visits from the researchers. Ten units asked for an additional supply of flash cards because of their popularity; seven units wanted extra training loose-leaf binders.

### Interprofessional medication review based on collegial mentoring

All units conducted the medication review. Some physicians had responsibility for several units and thus the visits were coordinated so that they could be performed in one appointment. Six units took the opportunity to have additional medication reviews. All patients were assessed prior to the scheduled medication review. The nursing home physician and a nurse from the unit attended each medication review; in addition, an extra nurse or the unit manager attended about half of these medication reviews. Using the COSMOS method, the group spent on average 1.5 h (range 1 to 2 h) performing medication reviews for eight patients. The first few patient cases were discussed extensively, while decisions were easier to make with the subsequent cases, because similar issues had already been discussed in previous cases.

### Implementation process

All units used the patient logs, though 57 patients (19%) were missing log entries over four months, attributable to either death or being moved from the unit during the intervention period (77%). As the total number of patients remaining in the study declined, the total number of patients changed throughout registration. For the first four weeks, the log was filled in for 211/294 (73%). The completion rates for week 8, 12, and 16 were: 206/288 (71%), 140/276 (51%), and 198/271 (73%), respectively. Of the patients with at least one entry in the logs, 220 (92%) had received a medication review. Medication indications were recorded for 200 (83%) of the patients. For 184 (77%) of the patients, either the patient himself/herself or relatives were informed about changes in drugs, and for 34 (14%) patients the nurse filling in the log did not know whether this information was given. In 72 (30%) of the cases, a drug was reinstated after a pause, and changes in health were documented in 204 (77%) of the patients (Table 4).

**Table 4:** Feedback by patient logs

	Whole period (*N* = 240)					
	Yes		No		Not applicable; don’t know	
Question in patient logs	N	%	N	%	N	%
Had at least one medication review	220	92%	16	7%	3	1%
Indication on each drug	200	83%	36	15%	4	2%
Informed patient and/or relative about change	204	85%	9	4%	26	11%
Reinstated drug after pause	72	30%	141	59%	27	11%
Documented change in patient health	184	77%	20	8%	34	14%

The answers in the patient logs were coded accordingly: If there were one or more “yes” in the 16 weeks of registration, the answer was coded yes. If there were one or more “no”, and no entries answered “yes”, we coded it as no. If there were one or more not applicable/don’t know, and no entries with yes or no, we coded it as not applicable/don’t know. The table includes only patients with at least one entry (57 excluded). Due to missing data, the numbers do not add up to 240

### Barriers and promotors for good implementation

Healthcare staff reported potential barriers and promotors for the implementation process during the medication review and the midway evaluation (Table 5). The staff welcomed this approach, which they felt, created an arena for learning, engagement and further development. One participant also expressed this enthusiastically: *I want to run back to my nursing home unit and look over all the medication charts right away!*

**Table 5:** Barriers and promotors for good implementation

Barriers	Promoters
New and difficult clinical instruments	Engagement
Lack of competence	Arena for learning
Practical challenges with changing drug regimes	Introducing a colleague to discuss difficult decisions with
Poor knowledge about electronic patient records	The intervention was perceived as important and relevant
Lack of time	Improved communication
Ethical dilemmas	Pleased relatives

The implementation of the systematic clinical evaluation by means of validated assessment instruments was straightforward for most of the nursing staff. Meanwhile, some units struggled with new or unfamiliar instruments and some of the nurses were less interested or did not regard the evaluation of the patient as their responsibility. Licensed practical nurses also felt less competent to evaluate changes in patient health or to communicate with the relatives in connection with the medical changes. The introduction of the word “pause” was regarded as a relief because it was easier for the relatives and the other staff in the unit to accept a pause rather than discontinuation of a drug. Practical difficulties where mainly related to the use of multi-dose dispensed drugs when several medications were changed at the same time. The doctors also had varying degrees of knowledge about the electronic patient record and some found it difficult to alter prescriptions. Thus, they depended on nurses to be present at the medication review and to follow up and document the changes in the system. Despite the fact that most GPs did not attend the seminar at baseline, the collegial monitoring was seen as positive and they were receptive to the medication review with the researchers. They appreciated the discussions with the nurses and researchers in the interprofessional setting. Not surprisingly, there was not always agreement in their professional opinions but observations made by the nurses in advance or related to scorings by the clinical assessments, influenced the prescribing routines positively and improved the communication between the staff members.

Several physicians suggested that the collegial support from the researchers and the interprofessional discussion provided help in the decision-making processes, as one colleague commented: *It is never easy to find the correct timing for deprescribing, for instance, anticoagulants to prevent a stroke. It is an ethical issue, you know.* Notably, these judgments were often related to drugs that were initiated during a hospitalization, often a long time ago, even when indications were no longer relevant.

Lack of time was a key barrier mentioned by all participants, including limited time for training, evaluation of the patients, and proper documentation. Meanwhile, motivated managers were able to initiate the medication review, despite the time-barriers. One nurse said: *Our boss encourages us and has provided a schedule for all the training and observations we are supposed to do; she also checks whether we have done it.* To conclude, participants felt that the intervention was needed and relevant. It granted knowledge and highlighted problems that they all felt were present. In addition, the relatives reported to the nurses that they were pleased with this thorough approach.

## Discussion

This study describes a new approach for systematic medication review based on clinical assessment of the patient and collegial mentoring of the physicians in Norwegian nursing homes. The implementation of the intervention was highly appreciated and well received by the physicians and created enthusiasm among nursing staff. The approach improved communication between the health personnel, patients, and the relatives. After four months, 92% of the patients had undergone a medication review, changes in the patients’ health were documented for 77% of the patients. Thirty percent of the patients were put back on a deprescribed drug. Lack of time was the most frequently reported barrier against the intervention as well as difficulties in engaging everyone in the unit. The systematic use of clinical assessment tools before and after the medication review was of key importance to the clinician because this facilitated the optimizing of safe prescribing patterns.

To our knowledge, this is the first study that describes the implementation strategy of collegial monitoring combined with thorough clinical testing of nursing home patients in connection with a medication review. Nursing home patients often suffer from neuropsychiatric symptoms, and consequently, psychotropic drugs are frequently prescribed [36]. To avoid unnecessary drug use, and thus side effects and interactions, it is a prerequisite to assess relevant clinical symptoms before and after treatment has been initiated. In an Australian randomized controlled trial, Potter and colleagues included 96 participants, who were systematically assessed by two researchers [12]. During the medication review, they were able to withdraw risk-modifying drugs, and to some extent symptom-modifying drugs. However, they did not report how neuropsychiatric symptoms were assessed and re-assessed after optimizing the medication lists. In the WHELD study, Ballard et al. focused on antipsychotic drug use in connection with neuropsychiatric symptoms on 187 nursing home patients [37]. After 9 months, the intervention group reduced antipsychotic drug use by 50% compared to the control group. The antipsychotic review group experienced a worse outcome of overall neuropsychiatric symptoms after the procedure. Meanwhile, social interaction and exercise proved to be essential to alleviate these symptoms. Both studies describe complex interventions but the implementation strategies were not carried out as planned in these settings. Furthermore, none of these studies included the nursing home physician in the medication reviews. In a different publication [38], we describe how the COSMOS study showed significant reduction in use of antihypertensive drugs in the intervention group compared to the control group, without any lasting effect on pulse or blood pressure.

It is of key importance to follow up and document changes after the medication review. We found that nearly 80% of the patients had changes in their health documented after the medication review. We also found that 30% had a drug reinstated after a pause. The study by Potter et al. had 41% unsuccessful withdrawal rate [12]. A Swedish study focusing on improving health monitoring of nursing home patients reduced drug use and increased documentation and follow-up [39].

The development, implementation and evaluation of complex interventions as described by the Medical Research Council guidance, UK, is challenging [40]. The process depends on a range of possible outcomes, the variability in the target population, and the number and content of the elements in the intervention package. Thus far, few trials have focused on the critical issue of whether the implementation of a systematic medication review is feasible in nursing homes or whether the staff would be receptive to the intervention [16]. Implementation studies allow for testing effectiveness of an intervention and at the same time investigating the implementation [41]. We found that the medication reviews were implemented in all wards and almost all patients received a medication review. In contrast, in the ARCHUS study from New Zealand, using education and multidisciplinary teams, only 23% of the participants in the intervention group were discussed during the team meetings [42].

Promotors and barriers affect the implementation process and depend on environmental factors, resources, beliefs about consequences, and social and professional roles [18]. In our study, time and available resources are considered the greatest barriers to implementation in clinical practice. However, some participants highlighted the value of prioritizing an intervention they perceived as being important, leading to increased knowledge among the healthcare professionals. A systematic review demonstrates that key factor for successful interventions in nursing homes are the involvement of the stakeholders and management-level healthcare professionals and to enlist commitment to support nursing staff to prioritize the intervention [43].

During the collegial mentoring, we encountered a number of ethical dilemmas when drugs or doses were changed or withdrawn. For instance, the deprescribing of anticoagulants or antibiotics often led to discussions about possible consequences. Interestingly, physicians tend to place more emphasis on actions than on omissions, and guilt deriving from negative consequences of an action is greater than guilt from inaction [44]. We observed that the process may create an arena for discussion and problem solving, and that it may bolster the communication between the participants. The roles of each participant in the medication review process were clearly defined. This might explain the feeling of improved communication between all the involved parties, leaning a voice in the interprofessional discussion to everybody.

In our study, we met nursing home staff members who were eager to expand their knowledge. Although physicians were often more difficult to include, commitment among staff members has been highlighted as a key factor for successful implementation, whereas a high turnover rate in personnel appears to weaken participation and implementation [45]. Despite the fact that the physician has the main responsibility for drug prescription, physicians have routinely been ignored in studies on medication reviews and only few studies include the attending physician [15–17, 24–26]. Meanwhile, in other countries, engaged pharmacists may assist in medication review. This was not possible in our study, because in-house pharmacists are seldom available in Norwegian nursing homes.

The main strengths of this study are the comprehensive sample size, the variety of units, and the active involvement of the patients’ physicians and nurses. However, this paper also has some limitations. It was not in the scope of the present paper to report patient-related health outcomes, or to evaluate changes in quality of life, as called for by Alldred et al. [15]. Our focus was to investigate the process in which the intervention was implemented as requested, which is often left out in other studies [16]. However, effect of the study has been reported elsewhere [20, 38]. The intervention might seem complex and time consuming, especially the assessment of patients’ pain and neuropsychiatric symptoms. Since these symptoms are common and drugs to treat these conditions are frequently prescribed, these assessments are essential. The use of two dedicated researchers in medication reviews is not feasible in clinical practice. On the other hand, we experienced during the medication reviews that it was the collegial discussion the physicians and nurses valued highly. We therefore suggest that local collegial networks might facilitate medication reviews. Unfortunately, we did not plan to use a structured assessment of the implementation Using a framework like RE-AIM would have strengthened the study [46]. Future studies may benefit from using such frameworks. The setting and variety of units makes the results generalizable and possible to compare across nursing homes in Norway and comparable countries.

## Conclusion

The medication review with collegial mentoring was implemented and well received in all units based on systematic assessments of patients for pain, neuropsychiatric symptoms, and health. Collegial mentoring was perceived as positive and valuable, and learning from each other was found to facilitate decision-making pertaining to difficult aspects of medication prescription. Knowledge about barriers and promotors can enhance the implementation of similar interventions.

## Abbreviations

ATC: Anatomical Chemical Therapeutic Index
CMAI: Cohen-Mansfield Agitation Inventory
COSMOS: Communication, systematic pain assessment and treatment, medication review, organization of activities, and safety
CSDD: Cornell scale for depression in dementia
FAST: Functional assessment staging
GP: General practitioner
MMSE: Mini mental state examination
MOBID 2: Mobilization-Observation-Behaviour-Intensity-Dementia 2 Pain Scale
NPI-NH: Neuropsychiatric Inventory- nursing home version
QUALID: Quality of life in late-stage dementia
SD: Standard deviation
START/STOPP: Screening tool of older persons potentially inappropriate prescriptions/screening tool to alert doctors to right treatment
